# Microstructure and mechanical properties of highly porous Hastelloy-X nickel superalloy produced by a space holder approach

**DOI:** 10.1038/s41598-024-84321-3

**Published:** 2025-01-02

**Authors:** Aleksandra Bętkowska, Marcin Podsiadło, Adelajda Polkowska, Grzegorz Włoch, Wojciech Polkowski

**Affiliations:** 1 Łukasiewicz Research Network, Krakow Institute of Technology, Zakopiańska 73 Str, Krakow, 30-418 Poland; 2 Faculty of Non-Ferrous Metals, AGH University of Science and Technology, Mickiewicza 30 Av, Krakow, 30-059 Poland

**Keywords:** Hastelloy-X, Nickel superalloys, Metallic porous materials, Space holder technique, X-ray computed tomography, Engineering, Materials science

## Abstract

Highly porous nickel-based superalloys appear as attractive candidates to be applied e.g. as seals in gas turbine engines instead of honeycomb structures. Among various methods of producing open-porous materials, a space holder approach provides number of benefits regarding economic and ecological aspects of production. In this work, the pioneering results of microstructure and mechanical properties analyses of highly porous Hastelloy-X nickel superalloy produced by the space holder approach, are presented. The materials were fabricated by using spherical fine Hastelloy-X powders and carbamide particles as batch materials. A multi-step powder metallurgy and thermomechanical processing was applied to produce open porous samples having a total volumetric porosity of 50, 60 and 70%. The produced materials were subjected to non-destructive (X-ray computed tomography) and metallographic inspections. Mechanical properties of the porous Hastelloy-X samples were examined in static room temperature compression tests, to discuss the effect of obtained porosity on compressive response.

## Introduction

Metallic porous materials (MPMs) are attractive candidates for both structural and functional applications. A high surface area and open porosity of these materials provide unique properties that are specifically attractive for lightweight automotive or space applications for example as filters, catalysts, thermal management devices, acoustic panels or energy absorption units^1,2^. Basically, the main feature controlling the overall performance of porous metals is a size and distribution of pores. In turn, the porosity arrangement in MPMs is controlled by an applied fabrication process and its variables. The manufacturing techniques of MPMs are based on liquid-state, solid-state or gas-liquid processes, while a selection of proper fabrication technology depends on the materials-based and application-oriented requirements. A solid-state space holder approach^3^ appears as attractive alternative to conventional liquid metal foaming techniques, because it allows easily controlling shape and size of pores inside the volume of processed component, under sustainable and cost-effective conditions. Furthermore, it should be underlined that the sustainability and cost-effectiveness of this approach comes from using:

- non-toxic, highly available and cheap materials as space holders;
- simple devices and relatively low energy consuming and safe processing conditions (cold compaction and pressure-less sintering in standard furnaces).

It is in a contrast to other approaches for producing porous metals, like a “polymer foam replica”^4^ technique or “gasar” technology^5^, that either provide a random distribution of pores and their size, or require special apparatuses operating under high gas pressure conditions (safety issues), respectively. Among various reported examples of MPMs (including these produced by the space holder approach) most of them are focused on aluminum, copper or specifically on biomedical titanium-based foams. On the other hand, there is much more limited information regarding processing and properties of high temperature, creep and oxidation resistant MPMs. This is mostly related to arising technological issues associated with high melting points and superior mechanical properties of materials predisposed for high temperature applications. These properties make them less prone to a pressure-less processing and compaction. A good example of that is porous nickel-based superalloys that are predicted to be potentially applicable as abradable seals materials in gas turbine engines. The European Commission-funded ADSEAL project^6^ provided important practical insights into the requirements for materials and structures in abradable seal technology for future devices. During the project, the same metallic alloy in the form of thin walled honeycombs, gradient fibre or hollow sphere structures, was examined in cyclic oxidation resistance and abradability tests. It has been documented that metal alloy hollow sphere structures combine very good oxidation resistance (that was superior or at least not worse than that of honeycombs) with the required abradability. Furthermore, it has been proposed that the functionality of hollow sphere structures might be further improved by reducing the sphere shell thickness and by increasing the sphere diameter.

These findings led us to initiate new research efforts focused on Hastelloy-X (H-X) nickel superalloy as a novel material in the field of porous high-temperature materials. The H-X is a Ni-Cr-Fe-Mo alloy that possesses a combination of very good oxidation resistance at temperatures as high as up to 1095 °C (exceeding that of Inconel 600, Alloy 625, Alloy 800 H), fabricability and high-temperature strength.It has also been found that the H-X alloy exhibits exceptionally resistance to stress corrosion cracking in petrochemical applications^7^. Therefore, these properties predispose the H-X alloy to be applied as a porous material for many hi-tech high temperature functional and structural applications. Unfortunately, there is a very limited information on fabrication, structure and properties of porous H-X materials. What is important, although very few previous reports about “porous” H-X alloys have been published, the scope, presented details and obtained results of these works are incomparable to these considered in the present paper.

For example, in the work by Molin et al.^8^, the total porosity of the examined H-X alloy was ∼25% (so, it was much lower than that in the present paper). Furthermore, there are no details presented about the fabrication process, but from the attached microscopic images it seems that the porosity has been produced by simply using a low-pressure sintering process. Therefore, the resulted structures were strongly inhomogeneous without having clearly defined shape and size of pores.

The other very recent related papers by Agnolin et al.^9,10^ present the results of using “commercial” porous Hastelloy-X alloy sinters as potential supports for membrane-assisted steam methane reforming. Once again, in these works there are no details shown about the fabrication process, but the provided microscopic images suggest that the porous Hastelloy-X samples have been prepared in the same way as that used by Molin et al.^8^.

On the other hand, these publications show that there is a considerable practical interest in utilizing bulk and surface properties of “porous” H-X alloy in even a greater number of engineering applications related to sustainable energy production and conversion (beside of these originally proposed for the aviation sector in or original manuscript). As the H-X alloy surpass most of superalloys in terms of performance properties, a successful implementation of this particular heat and creep resistance material, has got a special importance for many industrial branches.

Therefore, as opposite to the results reported in the literature, our work shows for the first time details on the fabrication process of highly porous Hastelloy-X alloy having pores with a spherical morphology and a size and distribution controlled by a space holder particles.

In this work, we present for the first time the results of our research on the fabrication of highly porous H-X alloys (with a volumetric porosity up to 70%). For this purpose, we used a multi-step powder metallurgy-based approach utilizing a space holder concept. The produced materials were subjected to both non-destructive and destructive structural characterization, as well as to room temperature compression tests.

## Materials and methods

### Fabrication of highly porous H-X alloys by the space holder approach

**Fig. 1:**
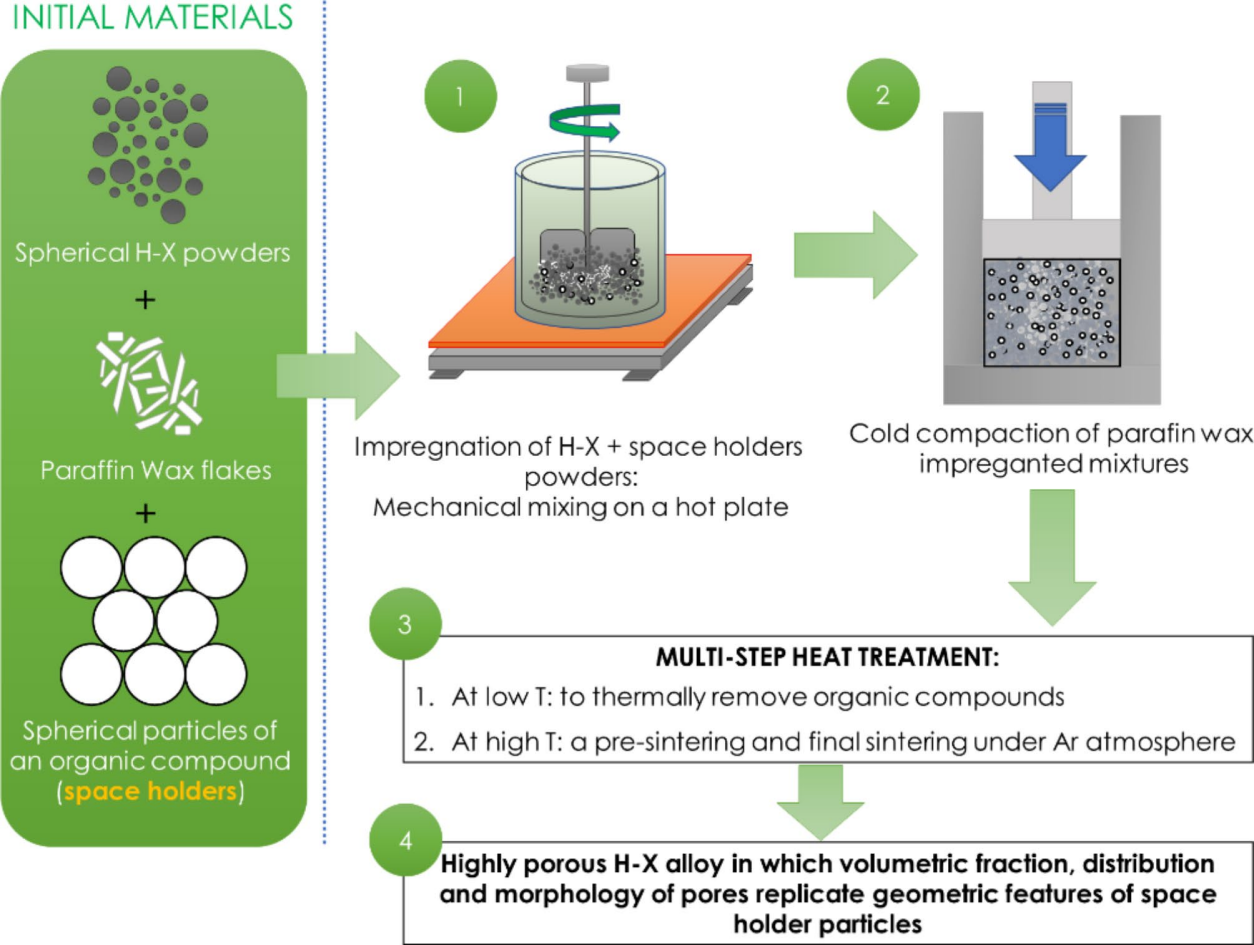
A scheme of the fabrication process of Hastelloy-X porous materials by a space holder approach.

Commercially gas-atomized spherical Hastelloy-X powders (Imphytek Powders, France) with diameters of D_10_ = 5.26 μm, D_50_ = 11.60 μm, D_90_ = 21.30 μm, were used as batch materials. The certified chemical composition of the powders was: Ni-22.1Cr-18.2Fe-9.2Mo-2.1Co (wt%). To fabricate highly porous H-X alloys, we adopted some technical solutions previously reported by Unver et al.^11^ for a fabrication of porous 625 Ni superalloy. A graphical representation of the entire process can be found in Fig. 1.

#### A preliminary screening of the variables for a development of the new space holder process for the H-X alloy

It must be underlined that although the general concept was adopted from the literature, a specific chemical composition and resulted properties of the H-X alloy required extensive preliminary research on the space holder-based process at each stage of its accomplishment. The following process variables were subjected to a preliminarily screening/examinations during the multi-variant technological trials, in order to achieve fully-compacted H-X porous materials:

(i) *a type of space holder material*: based on the literature data two candidates were pre-selected: a carbamide^11^ or CaCl_2_^12^, both showing a good dissolubility in water and/or ability to be thermally removed under predicted processing conditions;
(ii) *a fraction of the binder phase (paraffin wax)* between 3 and 5 vol%;
(iii) *temperature/time of the heat-treatment steps*: specifically, a temperature of the first stage annealing dedicated to a “gentle” removal of the space-holder particles was found to be crucial for the final results. Furthermore, temperature values applied upon the final sintering step (varied between 1200–1300 °C), gave important impact on the integrity of the porous H-X alloys.

**Fig. 2:**
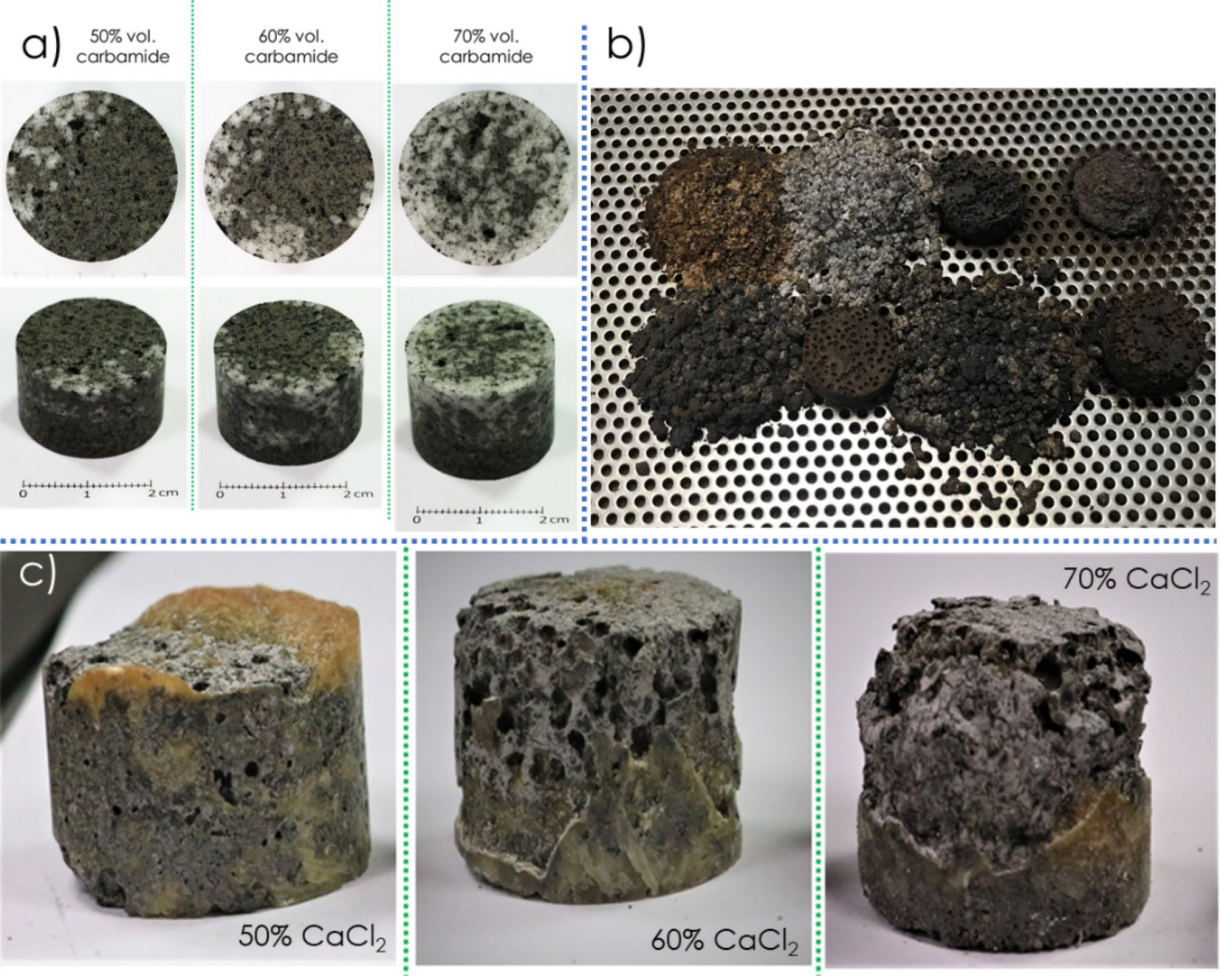
Examples of failed results of the preliminary research on the process development showing typical flaws such as an inhomogeneous distribution of space holder particles (**a**) and a complete disintegration (**b**) or a partial collapsing of pore structure (**c**).

Selected examples of failed preliminary trails are given in Fig. 2. Among them, an inhomogeneous distribution of space holder particles (Fig. 2a) and a complete disintegration (Fig. 2b) or a partial collapsing of pore structure (Fig. 2c), were observed as the most important flaws. By taking into account multi-variant nature of the process, it was not easy to indicate just only one reason for receiving unsuccessful final outputs. Nevertheless, based on the experimental results some undesired factors, were recognized and then modified/removed from the process. As we did use a pressure-less sintering (which is by the way, one of the advantages of the whole process) all of these efforts have been focused on supporting a diffusion-based joining mechanisms of H-X powder particles without collapsing the porous structure pre-formed upon the cold-pressing step.

Thus, during the preliminary trials carried out in a closed-loop, the process was optimized on many levels by:

- applying a proper amount of the binder phase;
- rejecting CaCl_2_ space holders;
- modifying the way of how the materials were mixed and put in a cold pressing die,
- adding new fixtures to heat treatment furnaces such as perforated plates or PTFE supports;
- setting proper temperature/time conditions of the annealing steps.

Finally, it allowed us to develop an optimized process described in details in the next subsection.

#### An experimental procedure applied for the optimized process

**Fig. 3:**
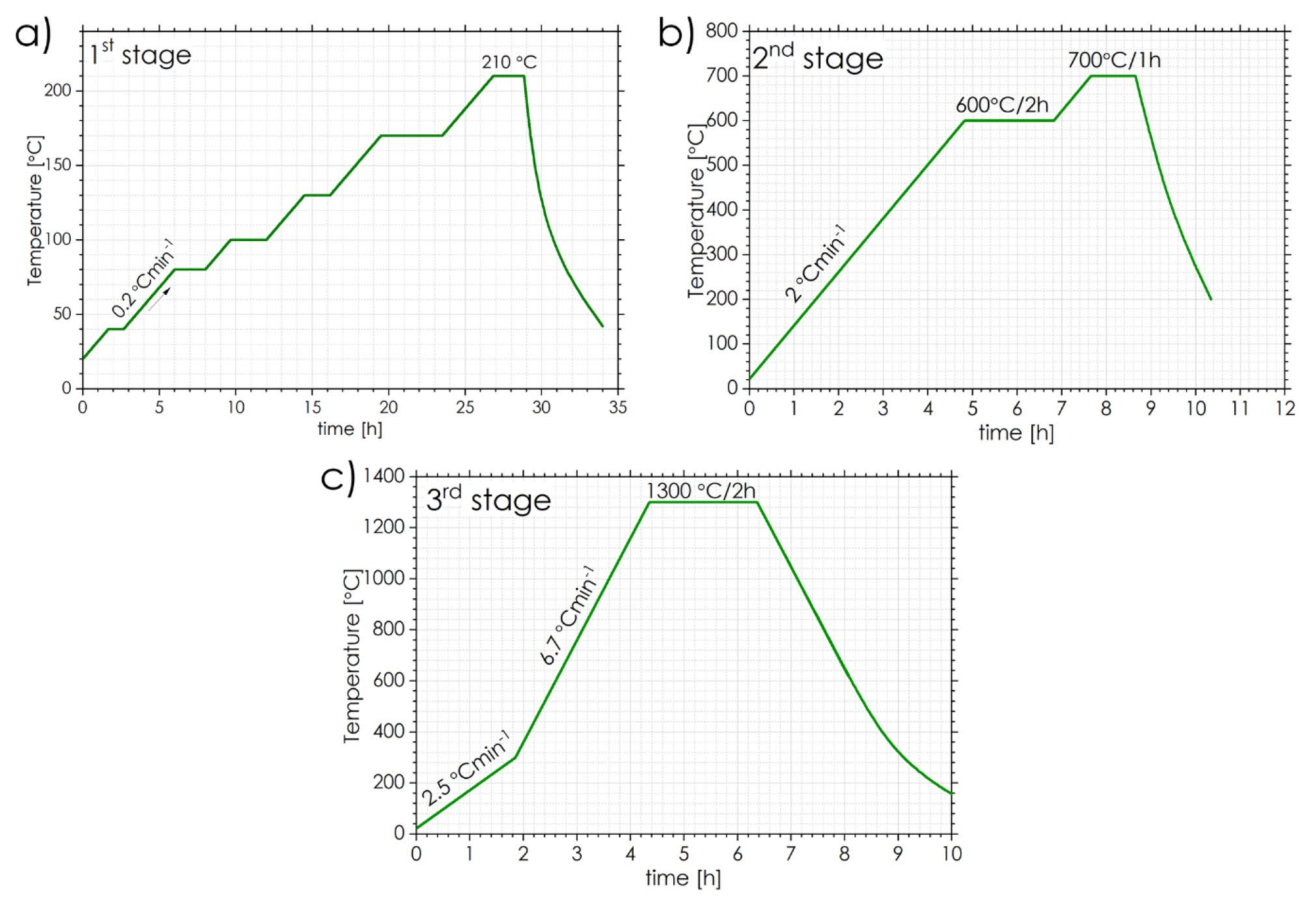
Temperature profiles for each stage of applied heat treatment: 1st stage—removing of organic and binder (**a**); 2nd stage—a preliminary sintering (**b**); 3rd stage—the final vacuum sintering (**c**).

The H-X powders were firstly mixed with a paraffin wax (3 wt%) and then mechanically stirred on a hot plate (90 °C). After that, spherical granules of carbamide (sieved down to a size of *d* = 2 –1 mm), were added and mixed together with wax-impregnated H-X powders. The following volumetric content of carbamide particles were applied: 50, 60 and 70 vol%. Next, the mixtures underwent cold compaction in a stainless steel die with a diameter of 23 mm under an isostatic pressure of 150 MPa. The cold compacted sinters were then subjected to a three-stage heat-treatment (Fig. 3). During the first stage, a slow heating up (at 0.2 °C min^− 1^) and annealing at 210 °C in air was applied to remove paraffin wax binder and carbamide particles. The second stage (annealing at 600 °C/2 h + 700 °C/1 h) was planned as a pressure-less preliminary sintering in argon flow atmosphere to complete the removal of organics. Finally, high temperature sintering at 1300 °C/2 h under vacuum of *p* = 10^− 2^ mbar, was applied to densify the compacts.

### Characterization of highly porous H-X alloys

The porous H-X alloys were subjected to a structural characterization by using non-destructive and metallographic techniques. The GE V|TOMEX|L-450 computed tomography (CT) device operating under voltage of 150 kV, current of 80 µA, exposure time of 250 ms and voxel size of 18.4 μm was used for the acquisition of X-ray images. Scanned objects were digitally reconstructed using Datos—x reconstruction and VGStudio Max 2.0 commercial software.

The microstructural characterization was carried out on cross-sectioned specimens by using light microscopy and scanning electron microscopy. FEI Scios™ Field Emission Gun Scanning Electron Microscope (FEG SEM) coupled with Energy Dispersive X-Ray Spectroscopy (EDS) and Electron Backscatter Diffraction system (EBSD), were utilized. Room temperature mechanical properties were examined in static compression tests carried out on in accordance to ASTM E9 standard cylindrical samples having dimensions of Φ = 6.7 mm × 10 mm. MTS 312.31 (200 kN) universal machine operating at a traverse speed rate of 0.005 min^− 1^, was applied. For each porosity three compression specimens were tested, and the average values of strength and strain parameters, were calculated.

## Results and discussion

**Fig. 4:**
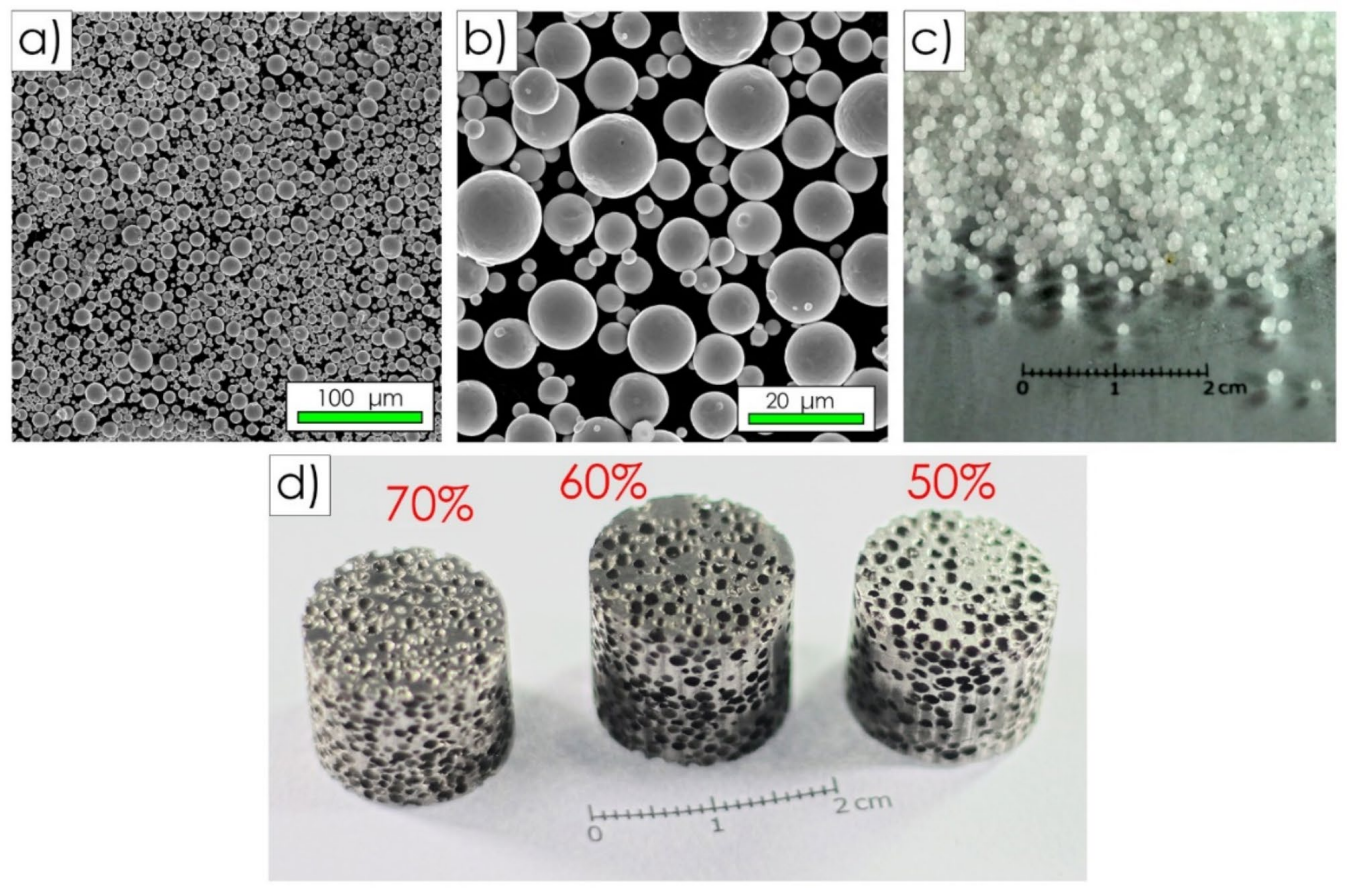
The materials used in the fabrication process: SEM images showing morphology of H-X powders used as the batch materials (**a**,**b**) and a macro view of the carbamide particles (**c**). An exemplary final H-X sinter having a porosity of 50, 60 or 70% (**d**).

The results of SEM inspections have confirmed a high sphericity and particle size of the commercial H-X superalloy powders that were used as the batch materials (Fig. 4a, b). As declared by the producers, the powder showed an average particle size below 15 μm. The powders were found to be free of shape defects (e.g. satellites). Spherical carbamide particles (Fig. 4c) combined with the applied processing allowed replicating their shape in the final porous samples. Finally, highly porous H-X sinters were successfully produced (Fig. 4d).

### Macro- and microstructure of porous H-X alloy

**Fig. 5:**
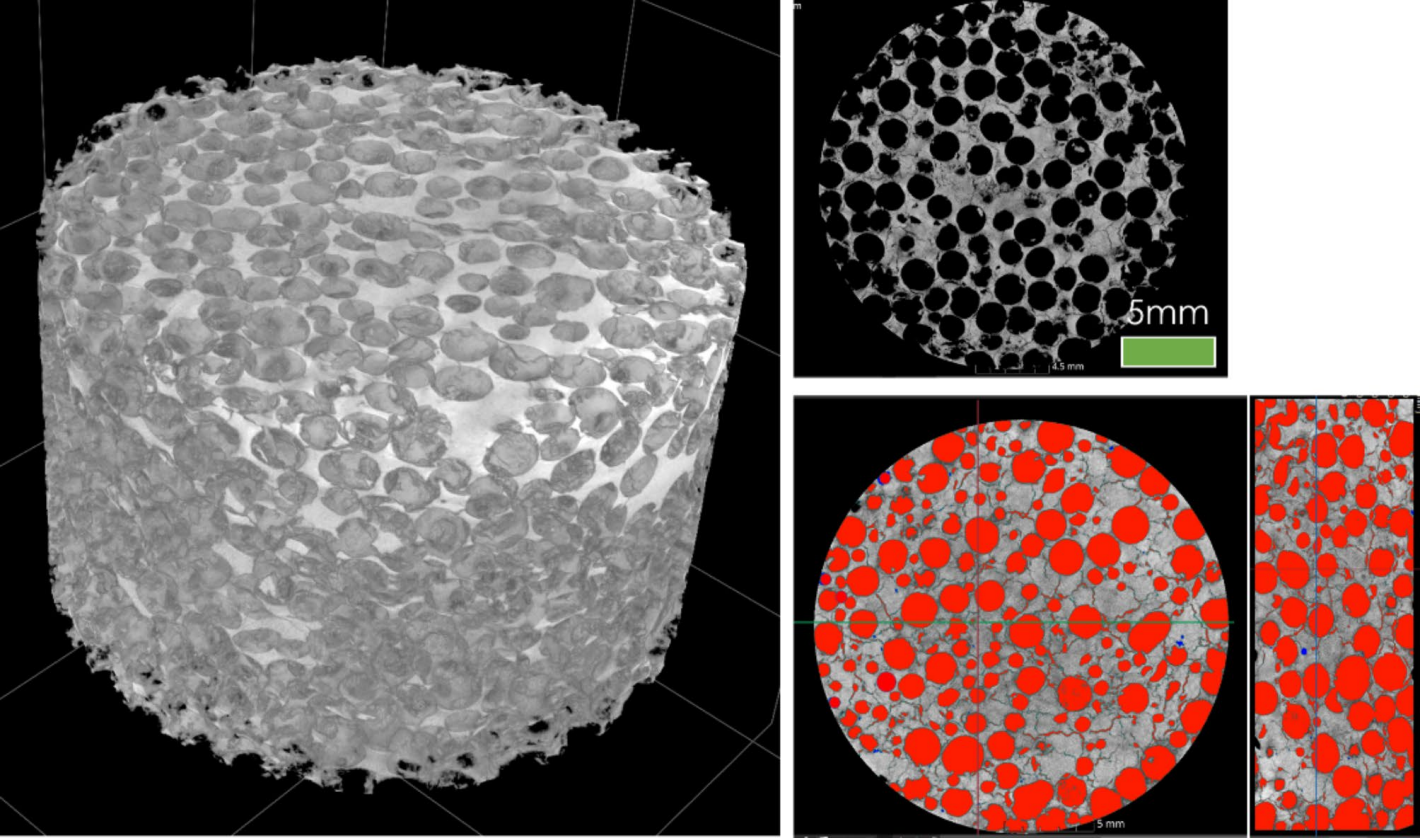
Exemplary results of CT inspections taken from the Hastelloy-X sample having a porosity of 70%.

**Fig. 6:**
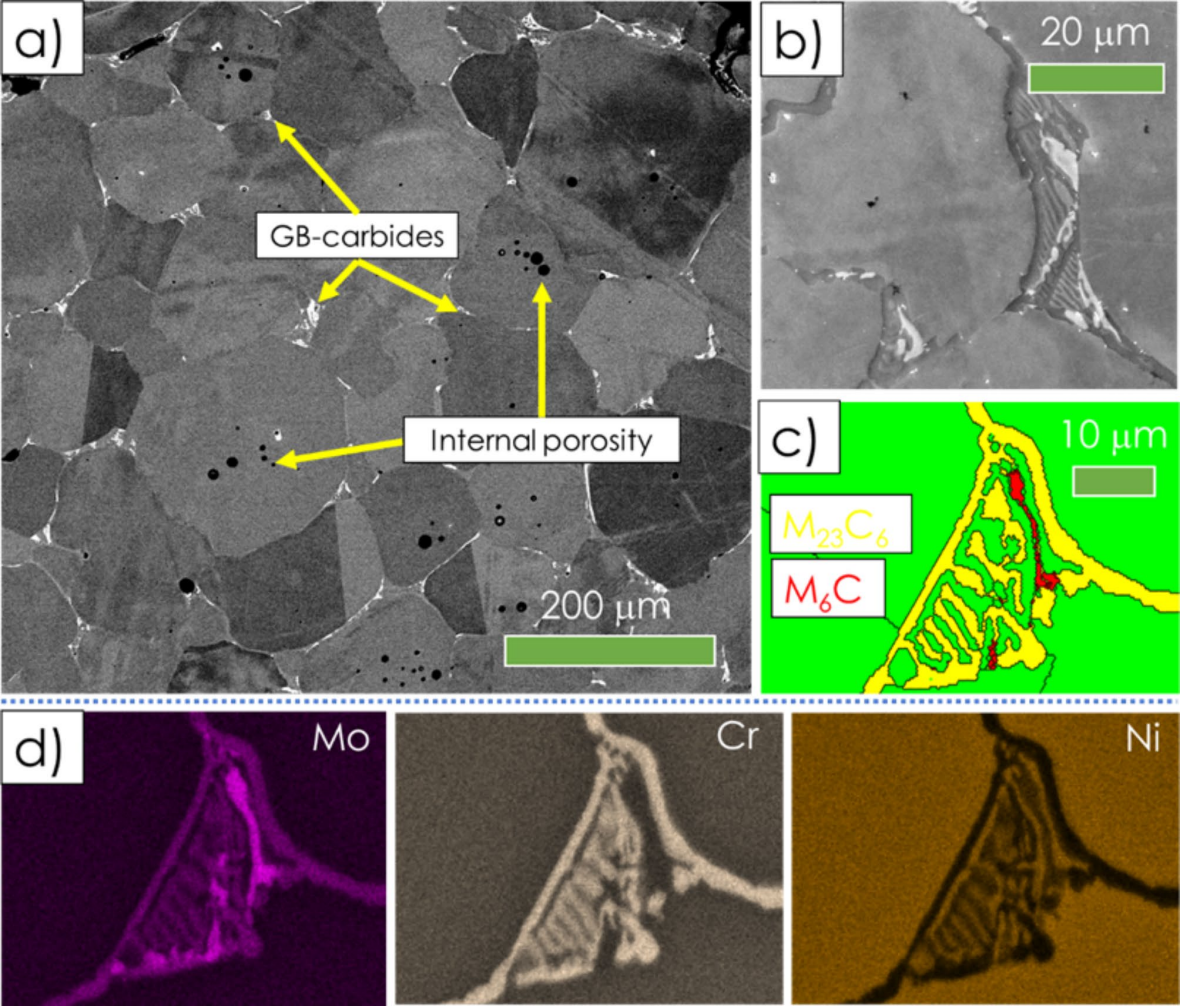
Selected results of the SEM/EDS/EBSD analyses of the Hastelloy-X sample having porosity of 50%: a microstructure of the wall (**a**); SEM/EBSD/EDS analyses of the grain boundary carbide precipitates (**b**–**d**).

The results of non-destructive CT analyses (see example in Fig. 5) revealed homogeneous distribution of porosity within the produced sinters. As revealed by the reconstructed models, even for a porosity as high as 70%, the sinters showed a good integrity and a well-defined porous structure. The shape and size of pores replicate the morphology of used carbamide particles. The results of metallographic analyses (Fig. 6) revealed: (i) an average grain size of ~ 120 μm: (ii) an existence of some internal microporosity (Fig. 6a); and (iii) a presence of skeleton-like precipitates at grain boundaries (GBs). More detailed analyses by simultaneous SEM/EDS/EBSD method (Fig. 6b–d) allowed recognizing these structural features as Cr-rich M_23_C_6_ and Mo-rich M_6_C carbides. The phase identification of GB precipitates is in line with results reported by Marchese et al.^13^, Lee et al.^14^ or Zhonggang et al.^15^ for additively manufactured Hastelloy-X alloy, having blocky like precipitates of the same kind along GBs.

On the other hand, a skeleton-like morphology of the GB carbides points towards a *discontinous precipitation* (DP) as the main governing reaction. The basic feature of this phenomenon is a “*lamellar*,* transformation product behind a GB advancing into a supersaturated matrix*”^16^. Over the years, the presence of DP-like products in heat treated nickel based superalloys has been documented and widely discussed by many authors^17^. Furthermore, we have also recently documented a presence of analogous structural features in another Ni-Fe-Cr-based alloy subjected to non-equilibrium oversaturation followed by aging treatments^18,19^, .

For the sake of discussing the results of our present work, the model recently proposed by Atrazhev et al.^20^ might be adopted. The authors have proposed that GBs mobility is the key factor governing the formation of either GB serration under low GBs mobility conditions or skeleton-type GBs structures (similar to these observed in the present work) when the mobility of grain boundaries is high. It is reasonable to assume that very high temperature applied during the final processing step (T = 1300 °C = 0.96*T*_*m*_), supports both high GBs mobility and diffusion kinetics. Therefore, it is proposed that the formation of skeleton-like M_23_C_6_/M_6_C products is driven by a local segregation of Cr to GBs areas combined with an effective grain growth (i.e. a migration of GBs) inside the matrix of produced porous sinters and a high chemical affinity of Mo to carbon. Furthermore, organic materials used during the fabrication process (paraffin wax and carbamide) can easily serve as the carbon source supporting the DP reaction.

### Room temperature mechanical properties

**Fig. 7:**
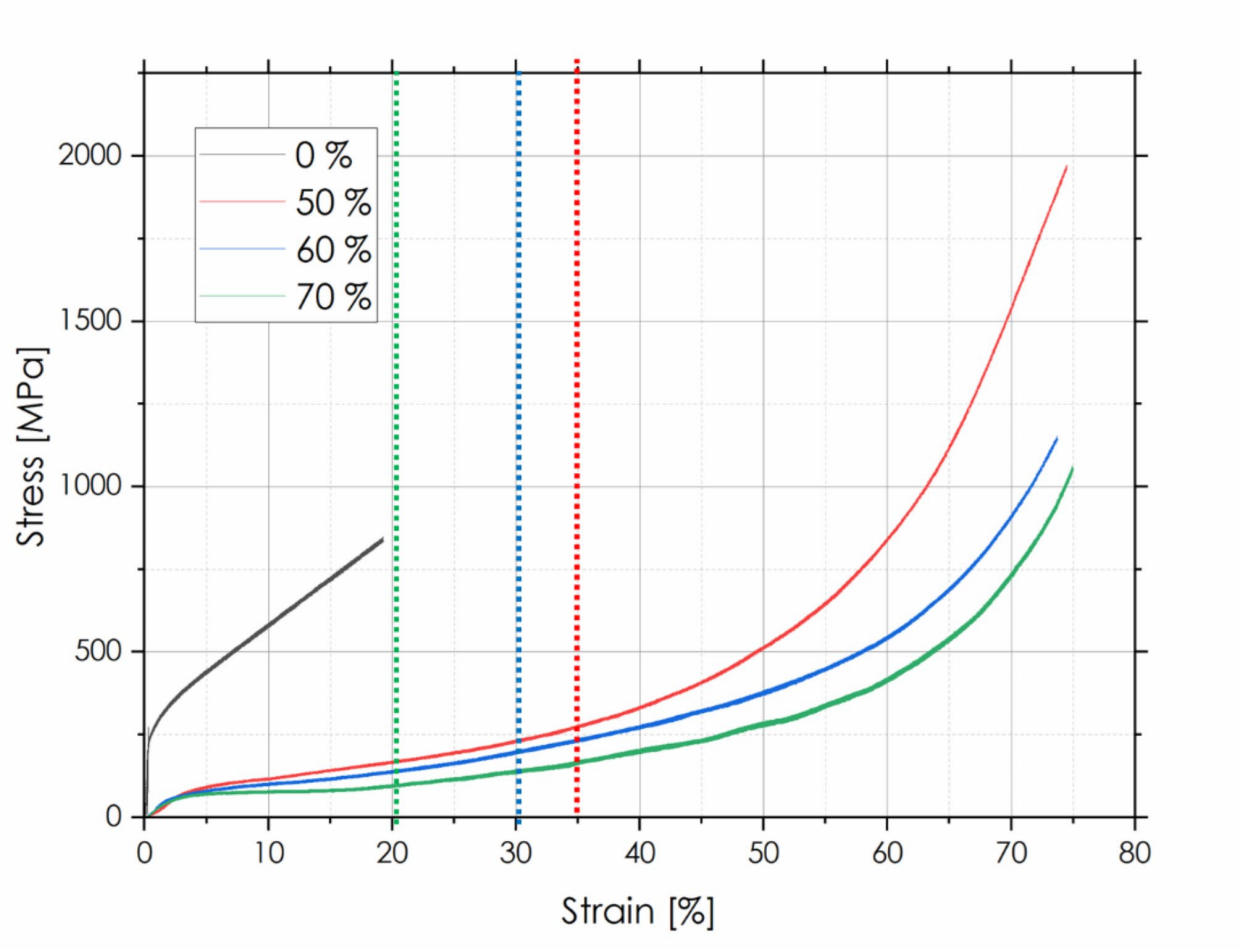
Compressive stress-strain curves of porous Hastelloy-X superalloy samples with 50–70% porosity (bulk alloy sample, marked as 0% was used as reference). Dotted lines denotes a transition between 1st and 2nd stage of deformation (details are given in the text).

Compressive stress-strain curves obtained for H-X samples with porosities of 50, 60 and 70%, are shown in Fig. 7, while values of quantitative parameters are listed in Table 1, and compared to literature data. The reference Hastelloy-X sample (marked as 0%) produced according to the same powder metallurgy-based procedure as described in the “A preliminary screening of the variables for a development of the new space holder process for the H-X alloy”, but without introducing porosity formers, was used for the sake of comparison.

The results obtained for the reference H-X alloy sample show a good agreement with these recently reported in the literature for the alloy processed by additive manufacturing techniques. However, it should be noted, that literature data regarding mechanical response of the AMed H-X alloy shows rather high scattering (Yield strength of 290–690 MPa; Ultimate Strength of 560–1060 MPa), as many variants of the processing and/or heat treatment, are applied in various laboratories.

On the other hand, it is worth noting that values available in the literature regarding mechanical properties of the Hastelloy-X alloy are mostly limited to those produce in tensile tests on additively manufactured specimens, and are rather highly scattered.

**Table 1:** The results of compression tests carried out on porous Hastelloy-X superalloy samples with 50–70% porosity (bulk alloy sample, marked as 0% was used as reference) and a comparison to reported literature data.

Porosity [%]	Yield strength (0.2) [MPa]	Ultimate strength [MPa]	Total strain [%]	Compressive stress [MPa]			
				at 5% strain	at 10% strain	at 25% strain	at 50% strain
Present work							
0	251*	847*	19*	445	583	n.a	n.a
50	73*	1965*	74*	96	116	195	511
60	61*	1144*	73*	82	100	163	375
70	54*	1043*	74*	69	75	115	280

References	Yield strength (0.2) [MPa]	Ultimate strength [MPa]	Total strain [%]	Remarks (processing, testing procedure)			
Literature							
^14^	294–324	566–796	5–57	Additive Manufacturing by Directed Energy Deposition and HIP post processing; tensile test			
^23^	558	845	30	Additive Manufacturing by selective laser melting and HIP post processing; tensile test			
^24^	680	910	13–20	Additive Manufacturing by selective laser melting - various processing conditions; tensile test			
^21^	350–610	630–790	9–45	Additive Manufacturing by selective laser melting; tensile test			
^25^	367	787	40	Wrought (hot forging), tensile test			
^26^	800	900	28	Additive Manufacturing by direct laser deposition and HIP post processing ; tensile test			
^22^	375–696	758–1067	19–51	Additive Manufacturing by selective laser melting; tensile test + heat treatments			
^27^	415–696	620–892	9–58	Additive Manufacturing by selective laser melting; tensile test			

*For each porosity three compression specimens were tested, and the average values of strength and strain parameters, were calculated. For all extracted parameters, scattering of the resulted values was not higher than 10%.

The results obtained for porous Hastelloy-X samples show typical effects of introducing a high volumetric content of porosity. With increasing porosity content in the Hastelloy-X alloy, the obtained curves became more and more “flattened”, i.e. a noticeable decrease of compressive strain was observed over the wide strain range. After reaching yielding point at a low level of ~ 50–75 MPa, the porous samples underwent a plastic deformation in two stages: (i) under a near linear-like course associated with a densification of sinters through deforming and breaking individual walls; and (ii) in non-linear regime characterized by a more intensive strain hardening. This destruction mechanism has been reported for example in the case of highly porous Fe-Al intermetallics^28^. It is found that a general strain hardening coefficient (expressed as a local slope of the compressive curve) decreases with the increment in the volumetric content of pores. This effect seems to be reasonable, as more pores in the sample volume, means a smaller load bearing cross-section area. Analogous mechanical behavior (and a similar shape of compressive curves and stress/strain values) has been previously reported by Unver et al. for porous 625 Ni superalloy^11^.

**Fig. 8:**
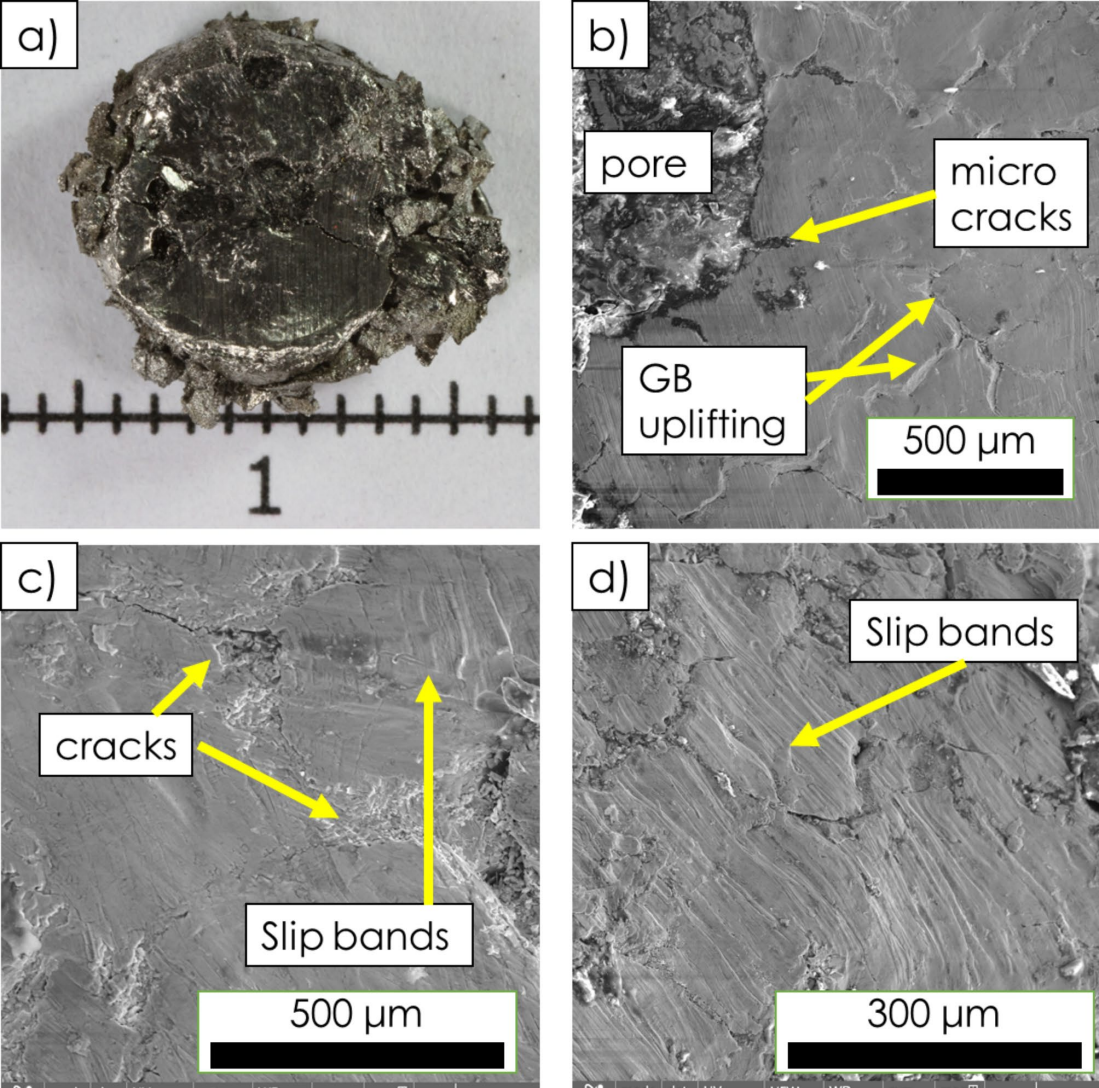
SEM fractographic inspections of porous Hastelloy-X superalloy sample (50% porosity) after the room temperature compression test: a macro view of crushed sample (**a**); closed-up views showing features related to plastic deformation and cracking inside cell walls, including grain boundary uplifting, slip bands and (micro)cracks (**b**–**d**).

The results of *post-mortem* SEM fractographic inspections (Fig. 8) carried out on the surface of compressed specimens revealed some features typically observed on fractures produced with involvement of an intensive plastic deformation. Specifically, a presence of “uplifts” along GBs (Fig. 8b) and slip bands (Fig. 8c,d) suggests that the final cracking was preceded by an accumulation of large plastic strain. Moreover, as some cracks were observed along grain boundaries, the role of DP products on mechanical properties ought to be further elucidated.

## Conclusions and future remarks

In this work, we show for the first time, the results of fabrication and characterization of highly porous (50–70 vol%) Hastelloy-X alloy. Based on the results of non-destructive and destructive structural characterization, compression tests and reported literature the following conclusions are written down:

1. The developed powder metallurgy-based space holder approach appears as feasible and sustainable method for producing highly porous Hastelloy-X alloy. The method involves non-toxic, cheap and easily available substances as binders and space holders, while the processing involves cold-compaction and pressure-less sintering steps.
2. Even for a porosity as high as 70%, the sinters show a good integrity and a well-defined porous structure. The shape and size of pores replicates the morphology of used carbamide particles.
3. The walls’ microstructure consists of an fcc matrix having the average grain size of ~ 120 μm: (ii) and a presence of skeleton-like precipitates at grain boundaries. The precipitates were recognized as M_23_C6/M_6_C products of the discontinuous precipitation reaction. Their impact on mechanical properties will be further evaluated.
4. The highly porous Hastelloy-X alloy shows prominently different mechanical behavior in compression tests, as compared to the bulk counterpart. Destruction of porous sinters takes place in two stages: densification of sinters trough deforming and breaking individual walls; and a more intensive strain hardening of desified speciemens. A general strain hardening coefficient (expressed as a local slope of the compressive curve) decreases with the increment in the volumetric content of pores.

Future planned works will be focused on examining application oriented performance properties. In this regards, mechanical strength, oxidation behavior and wear resistance of highly porous Hastelloy-X alloys at high temperarures, will be experimentally investigated.

## Data Availability

Related research data is available on demand from the corresponding author (at wojciech.polkowski@kit.lukasiewicz.gov.p).
